# Hypothalamus–Muscle Parallel Induction of Metabolic Pathways Following Physical Exercise

**DOI:** 10.3389/fnins.2022.897005

**Published:** 2022-07-19

**Authors:** Almog Katz, Meital Gonen, Yael Shahar, Asael Roichman, Batia Lerrer, Haim Yosef Cohen

**Affiliations:** The Mina & Everard Goodman Faculty of Life Sciences, The Sagol Center for Healthy Human Longevity, Bar-Ilan University, Ramat-Gan, Israel

**Keywords:** exercise, hypothalamus, hippocampus, muscle, metabolic pathways

## Abstract

The modern lifestyle requires less physical activity and skills during our daily routine, leading to multiple pathologies related to physical disabilities and energy accessibility. Thus, exploring the mechanisms underlying the metabolic regulation of exercise is crucial. Here, we characterized the effect of forced and voluntary endurance exercises on three key metabolic signaling pathways, sirtuins, AMPK, and mTOR, across several metabolic tissues in mice: brain, muscles, and liver. Both voluntary and forced exercises induced AMPK with higher intensity in the first. The comparison between those metabolic tissues revealed that the hypothalamus and the hippocampus, two brain parts, showed different metabolic signaling activities. Strikingly, despite the major differences in the physiology of muscles and hypothalamic tissues, the hypothalamus replicates the metabolic response of the muscle in response to physical exercise. Specifically, muscles and hypothalamic tissues showed an increase and a decrease in AMPK and mTOR signaling, respectively. Overall, this study reveals new insight into the relation between the hypothalamus and muscles, which enhances the coordination within the muscle–brain axis and potentially improves the systemic response to physical activity performance and delaying health inactivity disorders.

## Introduction

Physical activity refers to any level of activity that results from skeletal muscle activation, leading to a movement that is part of our daily lives. It has numerous health benefits, and its decline is associated with multiple pathologies. By contrast, physical exercise refers to planned, structured, repetitive activity. While in the past, physical activity was essential for human survival, modern life demands less movement and physical skills. Nevertheless, the physical inactivity of our daily routine has a major effect on our physical abilities, lifestyle, and energy accessibility. Thus, the importance of voluntary physical exercise, which can compensate for the decrease in physical abilities (i.e., gyms) (Morseth and Hopstock, 2020), and the need to study its influence have gained increasing relevance and are expected to provide better insights into physical inactivity disorders that lead to many damaging processes affecting the healthy life span (Hood et al., 2006; Coffey and Hawley, 2007; Egan and Zierath, 2013).

Exercise can be divided into two types of activities: endurance and resistance. Both are complex processes and influence whole-body energy homeostasis. They involve the activation of many tissues and organs at cellular and systemic levels and work in a synchronized manner (Joyner and Green, 2009; Hawley et al., 2014; Cartee et al., 2016). Since these activities require energy, it is highly important to understand the metabolic regulation of exercise and the effect of the latter on key metabolic pathways (Hawley et al., 2014). At least two key metabolic signaling pathways are involved in physical exercise, AMPK and mTOR (Bassel-Duby and Olson, 2006; Hood et al., 2006; Coffey and Hawley, 2007; Egan and Zierath, 2013). AMPK or mTOR induction is involved in endurance and resistance exercises, respectively. AMP-activated protein kinase (AMPK) is activated under limited energy supply by an increased AMP/ATP ratio, as in fasting (Kelly and Scarpulla, 2004; Akimoto et al., 2005; Lin et al., 2005; Jäger et al., 2007; McGee and Hargreaves, 2010). By contrast, mammalian target of rapamycin (mTOR) is activated under high nutrient availability, such as administration of amino acids, and promotes growth and protein synthesis, while inhibiting autophagy (Coffey et al., 2006; Ruas et al., 2012; Kittler et al., 2015; Ju et al., 2016). During exercise, there is an increased need for energy production; hence, ATP is essential to fuel cellular processes. Thus, during physical exercise, the aforementioned signaling pathways regulate gene expression, protein synthesis or degradation, and mitochondrial biogenesis to maintain cellular homeostasis (Lira et al., 2013; Hawley et al., 2014; Fan et al., 2015; Rocchi and He, 2017).

A significant amount of data was obtained describing the role of metabolic pathways in the skeletal muscles. Endurance exercise activates AMPK, which results in mTOR inhibition and mitochondrial biogenesis. AMPK directly activates PGC1α, which regulates nuclear genes that control mitochondrial biogenesis, such as nuclear respiratory factors (NRF)-1 and NRF-2. NRF-1 activates the mitochondrial transcription factor A (TFAM), which is translocated to the mitochondria and regulates mitochondrial DNA transcription. In skeletal muscles, PGC1α activity regulates mitochondrial biogenesis, which responds to neuromuscular input and results in muscle contraction activity (Kelly and Scarpulla, 2004; Hawley et al., 2006, 2014; Scarpulla, 2006; Cantó et al., 2015; Verdin, 2015).

mTOR activation is important for increased protein synthesis and induces muscle contraction in response to resistance exercise. mTOR exists as two distinct complexes: mTORC1 and mTORC2. However, our knowledge about the involvement of each complex in various exercises is limited. Insulin-like growth factor 1 (IGF-1) is a key upstream positive regulator of mTOR. The serine–threonine kinase AKT is a central regulator of multiple cellular processes, including cell proliferation, transcription, glucose metabolism, and apoptosis. IGF-1 positively regulates the increase in skeletal muscle mass *via* AKT-dependent mTOR phosphorylation, through the induction of protein synthesis. In turn, mTOR phosphorylates and activates the translational control proteins: ribosomal protein p70 S6 kinase (p70 S6K) and eukaryotic initiation factor (4E-BP1). Therefore, AKT activation leads to the activation of TORC1, which controls protein synthesis through the phosphorylation of p70 S6K and 4EBP1. The activation of p70 S6K leads to the phosphorylation of ribosomal protein S6 (pS6) and induction of protein synthesis (Coffey et al., 2006; Ruas et al., 2012; Kittler et al., 2015; Ju et al., 2016).

Autophagy is a highly conserved control mechanism which regulates the cellular response to stress, differentiation, growth, metabolism, and cellular homeostasis. Under the presence of stressors, such as physical exercise, autophagy is induced by the inactivation of the AKT-mTOR pathway and the activation of AMPK signaling pathway in skeletal muscles. The autophagy-dependent recycling of mitochondria promotes a mitochondrial biogenesis process (Lira et al., 2013; Fan et al., 2015; Ju et al., 2016; Rocchi and He, 2017). Thus, AMPK positively enhances mitochondrial biogenesis by increasing mitochondrial gene expression and the activation of autophagy, which are necessary for endurance exercise training (Hawley et al., 2014; Ju et al., 2016).

In contrast to the accumulated data on skeletal muscles, our knowledge regarding the regulation of metabolic pathways under physical exercise in other tissues, notably the brain, is poorly understood. Yet, some studies investigated the effect of exercise on hippocampal pathways that regulate processes related to cognitive functions, such as learning and memory, in addition to neuronal regulation of neurogenesis and synaptic plasticity. For example, exercise was found to improve dementia in individuals with Alzheimer's disease (Chen et al., 2016). During exercise, brain-derived neurotrophic factor (BDNF) reaches the neurogenic center in the hippocampus and activates cellular survival pathways, which are AKT-dependent. This induces transcription of genes responsible for almost all aspects of neuroplasticity through an increase in neurotransmission and BDNF-TrkB signaling, which enhance neuronal survival, synaptic plasticity, and neurogenesis (Park et al., 2013; Delezie and Handschin, 2018; Vecchio et al., 2018; Di Liegro et al., 2019).

During physical exercise, homeostasis is disrupted, and several organs and tissues work in synergy to restore the body's steady state. The hypothalamus is a critical brain region that connects the neuroendocrine system to physiological functions and regulates homeostasis. As the metabolic center of the brain, the hypothalamus plays a key function in the systemic regulation of metabolism. One of the essential factors activated under energy depletion is hypothalamic AMPK, which restores energy balance. This occurs by increasing glucose production and reducing whole-body thermogenesis, which decreases the energy output. Hence, physical exercise requires the rapid use and mobilization of energy substrates to modify changes in energy demands. However, to date, the effect of physical exercise on metabolism in the hypothalamus has not been described (Biran et al., 2015; Oh et al., 2016; Delezie and Handschin, 2018).

Here, we explored the effect of forced and voluntary endurance exercises on key metabolic pathways in the skeletal muscle, hippocampus, and hypothalamus. Interestingly, we found that despite the major differences in the physiology of muscles and hypothalamic tissues, a similar cascade of metabolic responses occurred in both. Thus, in addition to its key role in metabolism regulation, the hypothalamus replicates the metabolic response of the muscle.

## Results

### Voluntary Physical Exercise Has a Greater Effect on Body Composition Than Forced Exercise

**Figure 1:**
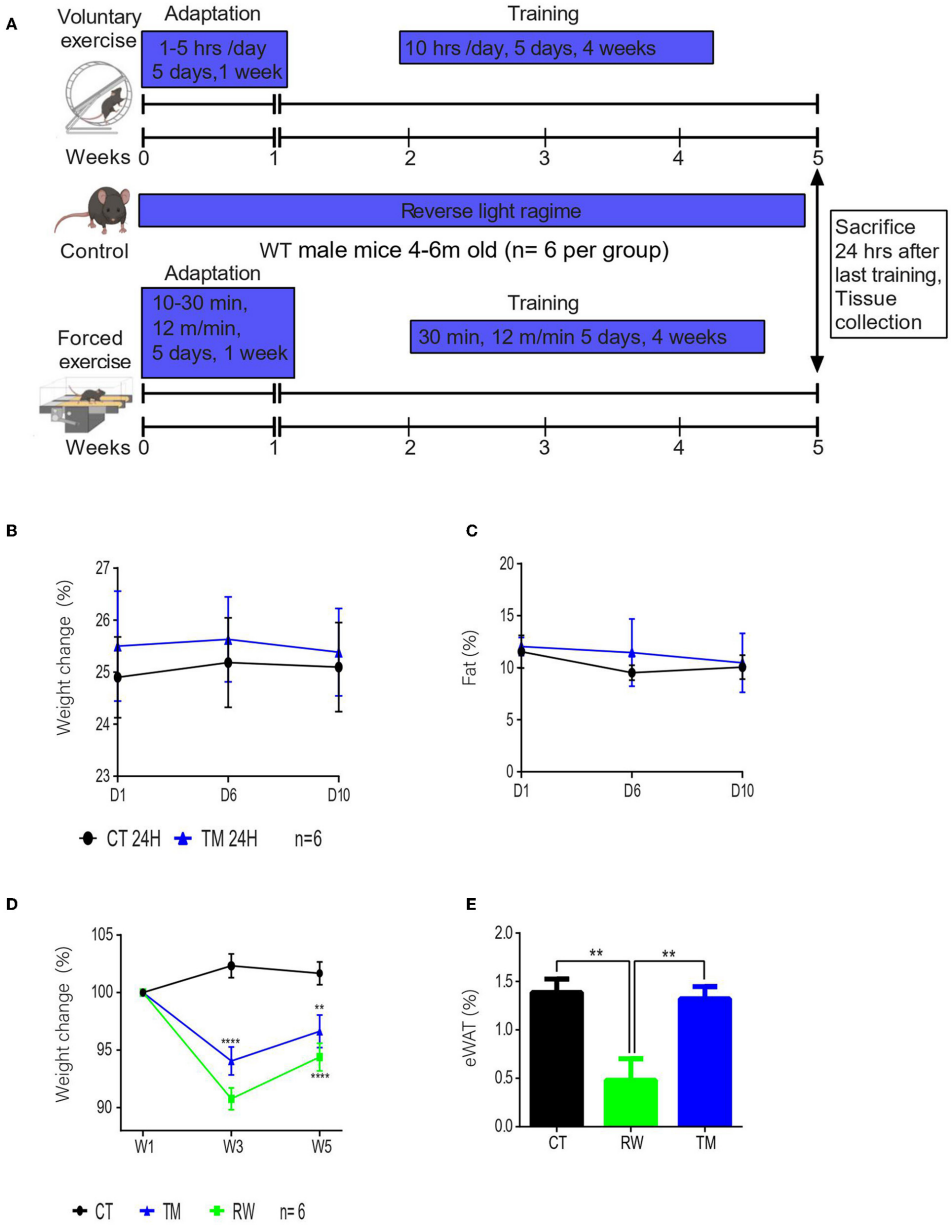
Voluntary physical exercise has a greater effect on body composition. **(A)** Schematic figure of experimental design. Drawn by Biorender. **(B)** Percentage change in body weight measurements before exercise (D1), on exercise day 6 (D6), and at the end of the training session, day 10 (D10). **(C)** Percentage average body fat of young (3 months) WT male mice from each group (control and treadmill). **(D)** Percentage change in body weight measurements, before exercise (start), after acclimation (W1), and once a week during exercise (W). **(E)** Epididymal WAT weight normalized to body weight at the end of the training session in young (4 months) WT male mice from each group control (CT), running wheel (RW), and treadmill (TM). The values shown are mean ± s.e.m.; ^*^p < 0.05; ^**^p < 0.01; ^***^p < 0.001; ^****^p < 0.0001 (two-way ANOVA, followed by Tukey *post hoc* test adjusted for multiple comparison for weight, and two-tailed *t*-test for fat (%); in **(A,B)** *n* = 6 for CT and TM; In **(C,D)** *n* = 6 for CT, RW, and TM.

To follow the physiological effect of endurance exercise, 4- to 6-month-old male mice were subjected to forced treadmill exercise (TM) or voluntary running wheel (RW) for 10 days or 5 weeks (Figure 1A; Supplementary Figure S1A). For short-term exercise, the body weight and composition of the TM group were measured after 10 days. In comparison to the control sedentary group (CT), after this short training, the TM mice exhibited similar weight and body composition (Figures 1B,C; Supplementary Figure S1B). In regard to long-term exercise (5 weeks), forced exercise resulted in significant weight loss (Figure 1D). Similarly, voluntary long-term running wheel (RW) exercise also led to significant weight loss (Figure 1D). Interestingly, RW, but not TM, endurance long-term exercise, resulted in a significant reduction in the body fat content (Figure 1E; Supplementary Figure S1C), thus suggesting that the reduction in weight in TM animals is due to the change in the non-eWAT fat content, for instance, in the muscle.

### Endurance Exercise Leads to Different Activation of AMPK and Sirtuin Signaling Pathways, Under Long-Term Forced and Voluntary Exercises in Gastrocnemius Muscles

**Figure 2:**
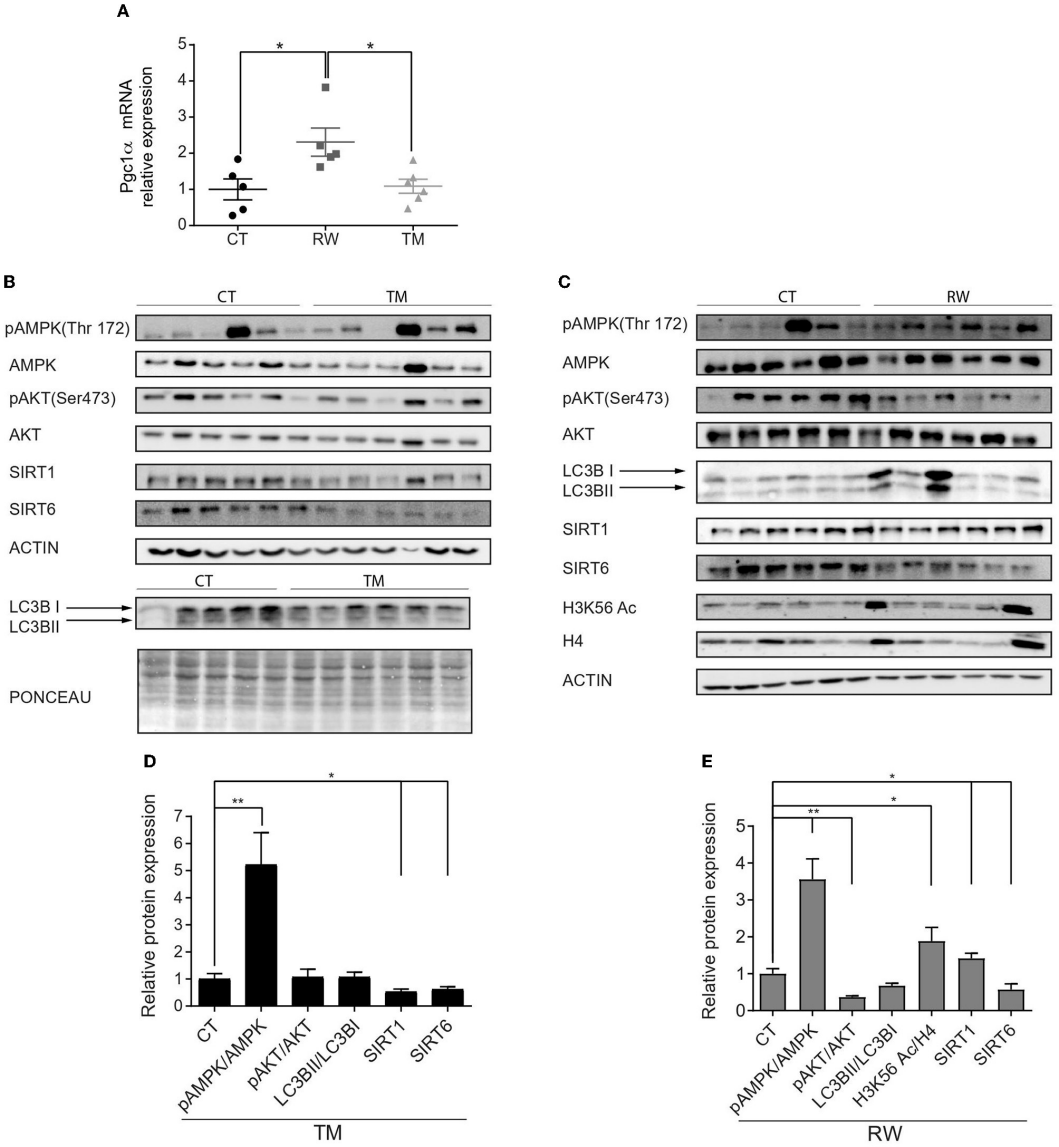
Activation of skeletal muscle AMPK signaling pathway is stronger under voluntary exercise. **(A)** Relative gene expression levels of *Pgc1*α. **(B,C)** Western blot analyses of pAMPK, AMPK, pAKT, AKT, SIRT1, SIRT6, H3K56Ac, and LC3B. **(D,E)** Densities of the bands were normalized to actin or Ponceau staining which served as an internal control in the gastrocnemius muscle of young (4-6 months) WT male mice. Ratios of phosphorylated AMPK to unphosphorylated AMPK, phosphorylated AKT to unphosphorylated AKT, LC3B II normalized to LC3B I, and SIRT1 and SIRT6 protein expression normalized to actin of each blot. H3K56Ac is normalized to H4. The values shown are mean ± s.e.m., *p < 0.05; **p < 0.01 (ordinary one-way ANOVA for **(A)**, two-tailed *t*-test for **(D,E)**; *n* = 5, 6 for CT, TM (black), and RW (gray).

Next, we further characterized the effect of endurance exercise on skeletal muscle metabolic pathways, including gene expression and protein level analyses. In comparison to the control group, no changes were found in gene expression levels of the key metabolic genes *Sirt1, Sirt6, Nrf-2*, and *tFAM* in gastrocnemius muscles (Supplementary Figures S2A–D) of either RW or TM exercise groups. In addition, the expression of metabolic genes did not change in response to short-term TM exercise in the quadriceps muscle (Supplementary Figure S2E). In accordance with previously published results, *Pgc1*α mRNA expression levels significantly increased in response to voluntary exercise (Figure 2A) (Hawley et al., 2014). To corroborate the RNA expression results, the protein levels of pAMPK, AMPK, pAKT, AKT, LC3B, SIRT1, SIRT6, and acetylated K56 of histone H3 (H3K56Ac) in skeletal muscles were measured at rest and under long forced and voluntary exercises (Figures 2B,C). In line with previous findings (Jäger et al., 2007), a significant increase in the pAMPK/AMPK ratio, representing AMPK activity, was found in both the RW and TM groups compared to the CT group (Figures 2D,E). Despite the increase in *Pgc1*α mRNA levels, in accordance with previously published results, following prolonged RW and TM exercises, the mtDNA content was decreased in the gastrocnemius muscles (Memme et al., 2021) (Supplementary Figure S2F). By contrast, the mtDNA content showed an upward trend in the quadriceps muscle following short-term TM exercise (Supplementary Figure S2G). In addition, no significant change was found in AKT activity, based on the pAKT/AKT ratio, between the TM and CT groups (Figure 2D). However, AKT activity was significantly reduced in the RW group compared to the CT group (Figure 2E).

AKT and AMPK negatively and positively regulate autophagy, respectively (Fan et al., 2015; Rocchi and He, 2017). However, in both RW and TM mice, no change in LC3B, an autophagy marker, was found (Figures 2D,E). In contrast to previously published results (Nemoto et al., 2005; Gerhart-Hines et al., 2007; Cantó et al., 2009; Guerra et al., 2010; Huang et al., 2016), mice performing TM, but not RW, exercise had a significant decrease in SIRT1 levels (Figures 2D,E). In comparison to the CT group, SIRT6 protein levels were significantly reduced in both forced and voluntary exercises (Figures 2D,E). Previously, we showed that SIRT6 inhibits AKT and activates AMPK (Kanfi et al., 2012; Roichman et al., 2016). Accordingly, a trend of an increased acetylation level of H3K56, a known SIRT6 substrate, was found in the RW exercise group compared to the control group (Figure 2E). The aforementioned proteins were also measured under short-term forced exercise and did not show any significant change (data not shown). Altogether, these findings suggest that RW training has a greater effect than TM activity on muscle metabolic pathways.

### Endurance Physical Exercise Leads to Activation of Both AMPK and AKT in the Hippocampal Region

**Figure 3:**
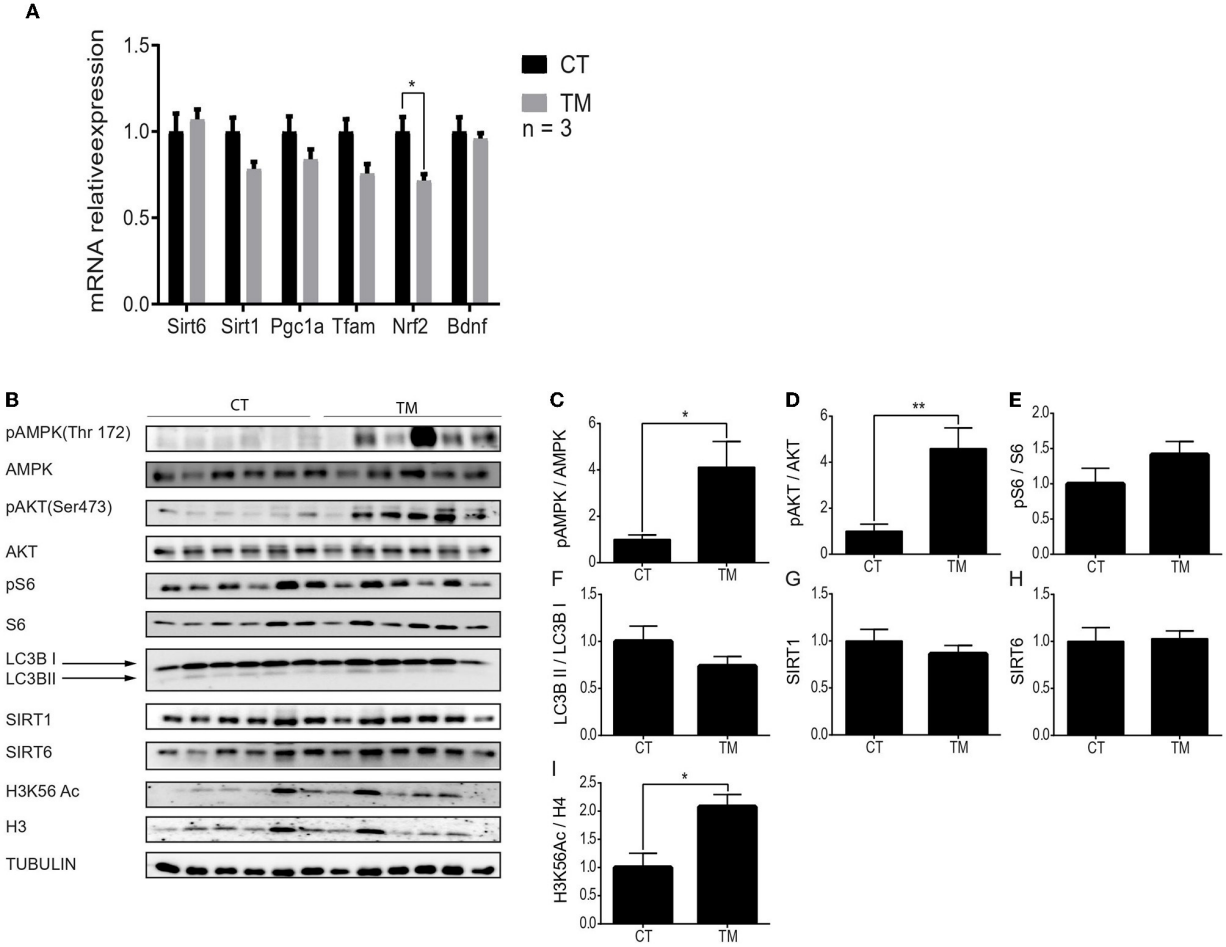
Unique hippocampal metabolic signaling pathway activation under forced exercise. **(A)** Relative gene expression levels of *Sirt6, Sirt1, Pgc1*α, *Tfam, Nrf2*, and *Bdnf* at rest (CT) or under short-term forced (TM) exercise. **(B)** Western blot analysis of p-AMPK, AMPK, pAKT, AKT, pS6, S6, LC3B, SIRT1, SIRT6, and H3K56Ac, in the hippocampi of young (4–6 months) WT male mice at rest (CT) or under long-term forced exercise (TM). Tubulin served as the loading control. **(C)** Expression ratios: Phosphorylated AMPK to unphosphorylated AMPK, **(D)** phosphorylated AKT to unphosphorylated AKT, **(E)** phosphorylated S6 to unphosphorylated S6, **(F)** LC3B II protein expression normalized to LC3B I protein expression, **(G)** SIRT1 and **(H)** SIRT6 protein expression normalized to tubulin, and **(I)** H3K56Ac normalized to H3 in young (4–6 months) WT male mice in the hippocampus. The values shown are mean ± s.e.m., *p < 0.05 (two-way ANOVA, followed by Bonferroni *post hoc* test in **(A)**, two-tailed *t*-test for **(C–I)**; in **(A)**, *n* = 3 for CT and TM; In **(B–I)** *n* = 6 for CT and TM.

Our knowledge regarding the brain's metabolic response to physical activity is limited. Thus, we focused on the response of the hippocampus and the hypothalamus to forced and voluntary physical exercises. First, gene expression levels were measured to assess the similarity between the effects of endurance exercise on hippocampal and skeletal muscle metabolic pathways. In comparison to the control group, *Sirt1, Sirt6, Pgc1*α, *Tfam*, and *Bdnf* gene expression levels did not significantly change after TM for 10 days (Figure 3A). Interestingly, the mRNA expression levels of *Nrf2* significantly decreased in the TM exercise group compared to the control (Figure 3A).

**Figure 4:**
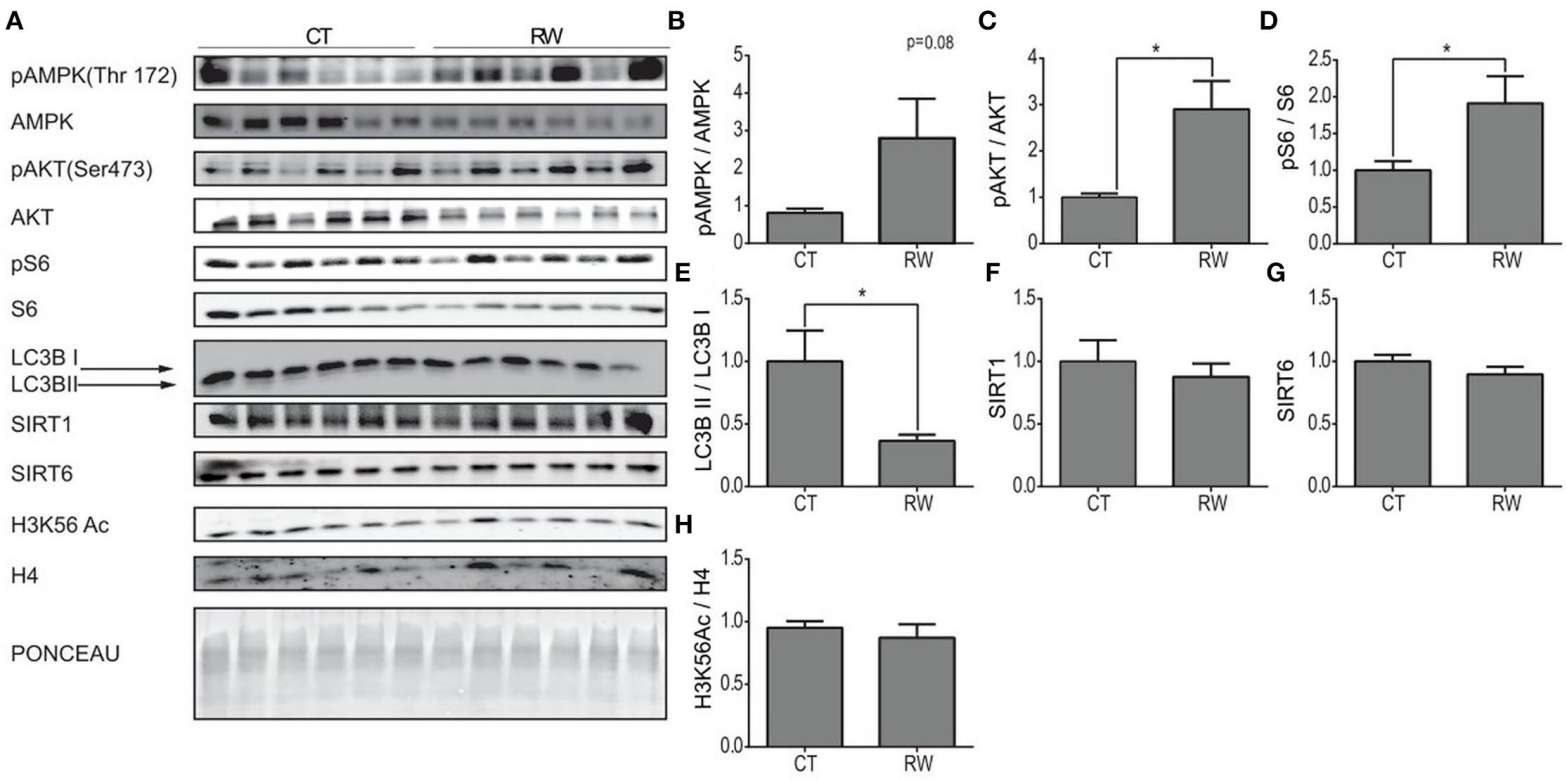
Unique hippocampal AMPK and AKT signaling pathway activation under voluntary exercise. **(A)** Western blot analysis of pAMPK, AMPK, pAKT, AKT, pS6, S6, LC3B, SIRT1, SIRT6, and H3K56Ac, in the hippocampi of young (4–6 months) WT male mice, at rest (CT) or under long-term voluntary exercise (RW). Ponceau staining served as loading control. **(B)** Phosphorylated AMPK to unphosphorylated AMPK ratio **(C)** phosphorylated AKT to unphosphorylated AKT ratio. **(D)** Phosphorylated S6 normalized to unphosphorylated S6; **(E)** LC3B II protein expression normalized to LC3B I protein expression. **(F)** SIRT1 and **(G)** SIRT6 protein expression levels normalized to actin. **(H)** H3K56Ac normalized to H4. The values shown are mean ± s.e.m., *p < 0.05 (two-tailed *t*-test); in **(A–H)** *n* = 6 for CT and RW.

Next, protein expression levels of pAMPK, AMPK, pAKT, AKT, pS6, S6, LC3B, SIRT1, SIRT6, and H3K56Ac were measured at rest and following long forced or voluntary exercise (Figures 3B, 4A). As previously demonstrated, protein expression levels of pAKTser473 are significantly increased in the hippocampus of TM and RW exercised animals compared to the CT group (Figures 3D, 4C) (Aguiar et al., 2011; Azimi et al., 2018). Yet, pS6 levels are significantly increased only in the RW exercise group compared to the CT (Figure 4D). In line with this observation, the LC3BII/LC3BI ratio, which is negatively regulated by mTOR, was also significantly reduced in RW mice (Figure 4E). Surprisingly, the phosphorylation levels of AMPK, which negatively regulates the mTOR-pS6 pathway, were increased in the TM and RW exercises compared to the control group (Figures 3C, 4B). Therefore, the significant AMPK activation in TM only may explain the lack of mTOR target, S6 and LC3BII, responses in this procedure.

Both SIRT1 and SIRT6 protein levels in the hippocampus remained unchanged in response to TM or RW exercise (Figures 3G,H, 4F,G). The acetylation levels of H3K56 increased in the TM group (Figure 3I) but did not change in the RW group (Figure 4H) compared to the control group. The same proteins were measured under short-term forced exercise but did not show any significant change (data not shown). Overall, these results may indicate that AMPK activity and its downstream pathway in the hippocampus are regulated by a different factor(s), other than SIRT6 and SIRT1, under physical exercise. In addition, these findings suggest that although AKT and AMPK have opposing activities, they could lead to the same effect in the hippocampus (Aguiar et al., 2011; Azimi et al., 2018).

### The Hypothalamic Region Shows the Same Signaling Pathway Activation Seen in Skeletal Muscles Under Forced and Voluntary Exercises

**Figure 5:**
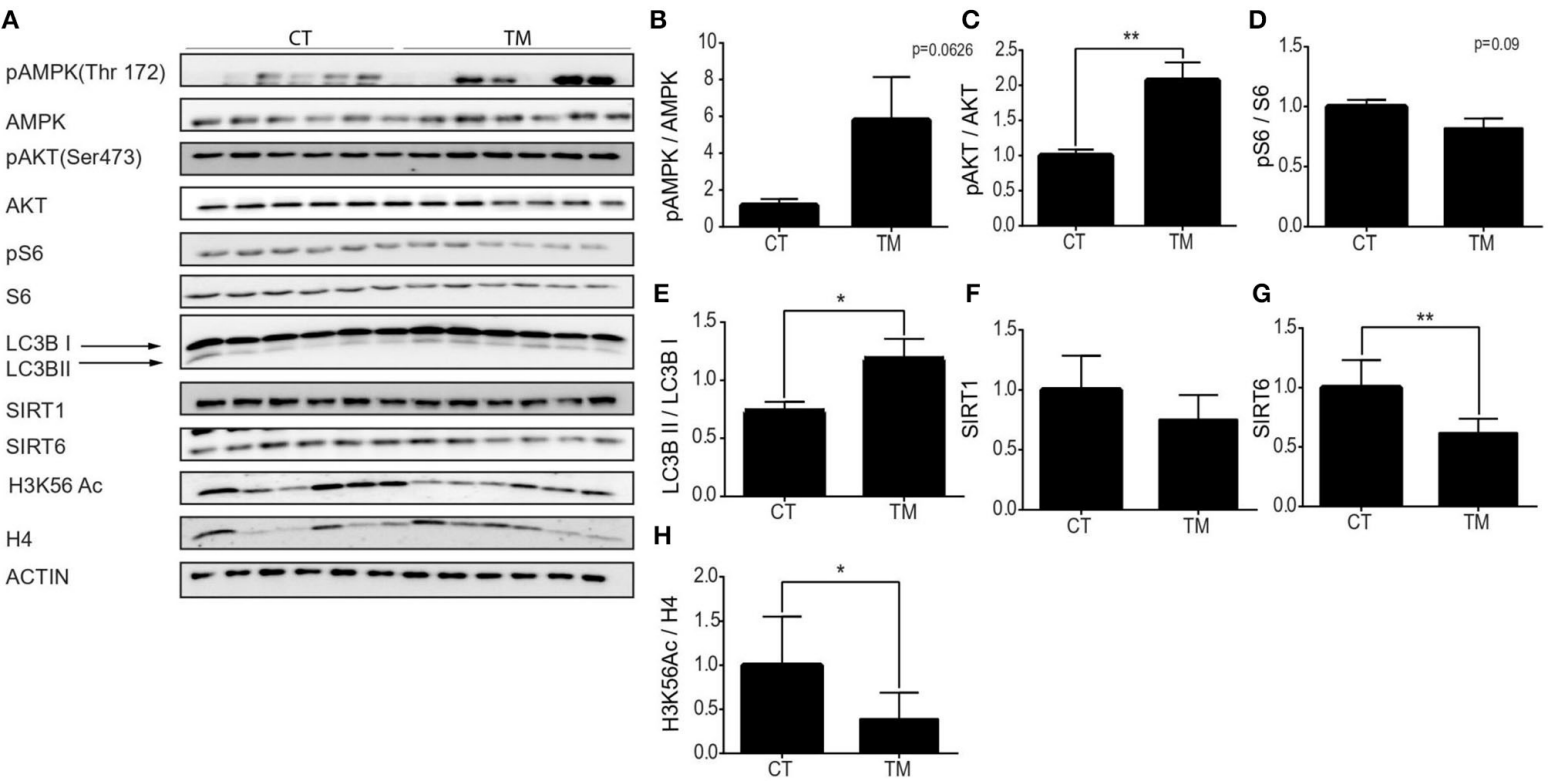
Forced exercise activates AMPK and SIRT6 pathways in the hypothalamic region. **(A)** Representative Western blot analysis of p-AMPK, AMPK, pAKT, AKT, p-S6, S6, LC3B, SIRT1, SIRT6, and H3K56Ac in young (4 months) WT male mice in the hypothalamus, at rest (CT) or under long-term forced exercise (TM). Densities of the bands were normalized to actin, which served as an internal control. **(B)** Phosphorylated AMPK vs. unphosphorylated AMPK. **(C)** Phosphorylated AKT normalized to unphosphorylated AKT. **(D)** Phosphorylated S6 normalized to unphosphorylated S6. **(E)** LC3B II protein expression normalized to LC3B I protein expression. **(F)** SIRT1 and **(G)** SIRT6 protein expression normalized to actin. **(H)** H3K56Ac normalized to H4 in young H4 in young (4 months) WT male mice in the hypothalamus. The values shown are mean ± s.e.m., *p < 0.05, **p < 0.01; (two-tailed *t*-test). In **(A–H)** *n* = 6.

**Figure 6:**
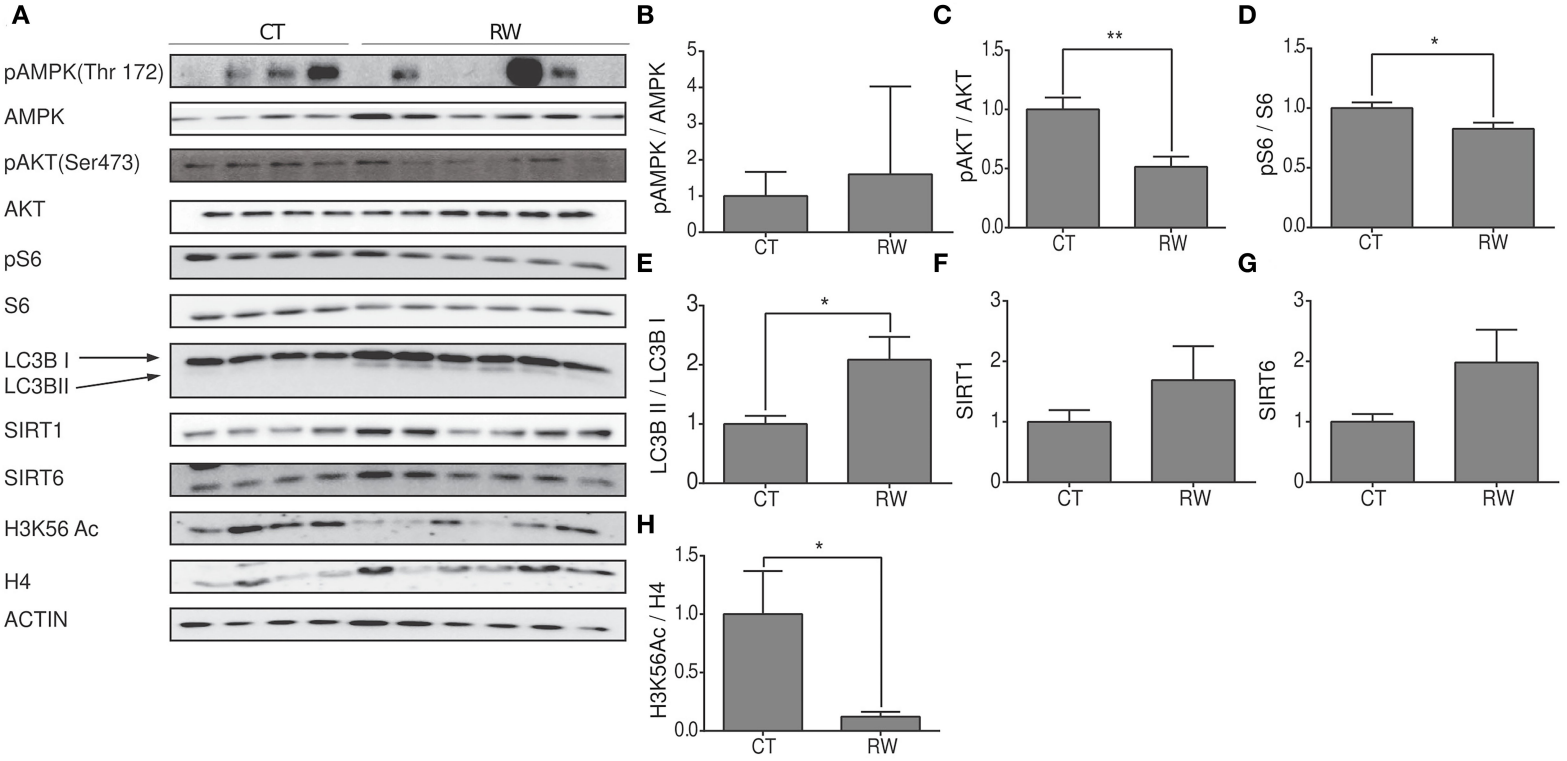
Voluntary exercise activates AMPK, SIRT6, and autophagy in the hypothalamic region. **(A)** Representative Western blot analysis of p-AMPK, AMPK, pAKT, AKT, p-S6, S6, LC3B, SIRT1, SIRT6, and H3K56Ac in young (4 months) WT male mice in the hypothalamus at rest (CT) or under long-term voluntary exercise (RW). Densities of the bands were normalized to actin, which served as an internal control. **(B)** Phosphorylated AMPK normalized to unphosphorylated AMPK; **(C)** phosphorylated AKT normalized to unphosphorylated AKT. **(D)** Phosphorylated S6 normalized to unphosphorylated S6. **(E)** LC3B II protein expression normalized to LC3B I protein expression. **(F)** SIRT1 and **(G)** SIRT6 protein expression normalized to actin. **(H)** H3K56Ac normalized to H4 in the hypothalamus of young H4 in young (4-month-old) WT male mice. The values shown are mean ± s.e.m., ^*^p < 0.05, ^**^p < 0.01; (two-tailed *t*-test). In **(A–H)** *n* = 4 for CT and *n* = 6 RW.

As mentioned before, our knowledge regarding the effect of exercise on hypothalamic metabolic pathways is limited. Thus, hypothalamic protein levels of SIRT6, SIRT1, AMPK, pAMPK, AKT, pAKT, pS6, S6 H3K56Ac, and LC3B were measured at rest, or following forced and voluntary exercises (Figures 5A–H). Hypothalamic AMPK activity is known to increase in response to reduced energetic levels (Huynh et al., 2016). Physical exercise mimics a low energetic status in comparison to the resting state (Huynh et al., 2016; Fujikawa et al., 2017; Ibeas et al., 2021), and accordingly, the pAMPK/AMPK ratio is increased in response to forced and voluntary exercises (Figures 5B, 6B). AKT is a positive regulator of mTOR. Interestingly, in comparison to the control group, the pAKT/AKT ratio showed a significant increase in the TM group (Figure 5C) with a significant decrease in the RW group (Figure 6C). In addition, in line with reduced levels of mTOR under low-energy conditions (Huynh et al., 2016), pS6 levels showed a trend toward a decrease in the TM group and a significant decrease in the RW group compared to the control group (Figures 5D, 6D). Furthermore, in line with pS6, in comparison to CT, the levels of LC3BII showed a trend of increased levels and a significant increase in the TM and RW groups, respectively (Figures 5E, 6E).

Both enzymatic activity and protein levels of SIRT1 increased in skeletal muscles of young rats and decreased in aged rats undergoing training (Koltai et al., 2010; Satoh et al., 2010). Interestingly, in young mice, in comparison to control mice, hypothalamic levels of SIRT1 were unchanged in response to forced or voluntary exercise (Figures 5F, 6F). The levels of SIRT6 were measured after exercise, as well. Surprisingly, SIRT6 protein levels decreased in the TM group (Figure 5G), while showing a trend toward an increase in the RW group (Figure 6G). To further examine SIRT6 activity, acetylation levels of H3K56Ac were measured. Both voluntary and forced exercises induced a significant decrease in H3K56Ac levels, indicating an increased activity of SIRT6 (Figures 5H, 6H). Altogether, with the exception of AKT activity in the TM group, these findings show that forced and voluntary activities mimic the hypothalamic metabolic cascade induced by the low energetic status.

## Discussion

Before this study, our knowledge regarding the brain's metabolic response to exercise was partial. Therefore, the metabolic signaling pathway that is responding to exercise was compared between skeletal muscles and specific brain regions. In the muscles, we found that the AMPK pathway was upregulated in both RW and TM exercise modes. In both exercise modes, SIRT6 levels were reduced, followed by an increase in H3K56Ac in RW. Uniquely, AKT activity was repressed, and SIRT1 and PGC1α expression levels were increased in RW, whereas SIRT1 levels were reduced in TM. In comparison to these responses, the hippocampus showed AMPK and AKT activation along with mTOR activation as manifested by higher pS6 and reduced autophagy markers in RW exercise. In addition, no change in SIRT1 and SIRT6, despite an increased H3K56ac level, was observed. After TM training, the hippocampal responses were identical to those found after RW, other than the effect on the mTOR pathway. Strikingly, in RW-trained animals, the hypothalamic AMPK-AKT-mTOR signaling pathway showed similar response to the gastrocnemius muscle's response. This was expressed by reduced pS6 and increased LC3BII. However, in contrast to muscle tissues, the SIRT6-H3K56Ac cascade was activated in the hypothalamus. Following TM training, both hypothalamic AMPK and AKT increased, but not mTOR. SIRT6 levels decreased, whereas the levels of its substrate, H3K56Ac, were also decreased. Altogether, as in the muscles, hypothalamic and hippocampal responses are unique to each exercise type. Importantly, direct comparison between the two types of physical exercises is not accurate specifically as they have different intensities and durations. Therefore, our comparison is between tissues within each exercise. Yet, in both types of exercise, the hypothalamic, but not the hippocampal, metabolic signaling response recapitulates at even stronger levels than the muscle response to exercise. These results suggest a muscle–hypothalamus synchronization response to physical exercise.

What accounts for the strong differences between the hypothalamic and hippocampal responses to exercise (Figures 4, 5)? Potentially, the difference in the role of AKT and its downstream target S6 in each brain region can explain the different responses to exercise observed between the centers. The hippocampus is the cognitive and memory center in the brain (Bird and Burgess, 2008). Here, AKT is involved in regulating signaling pathways related to neuronal survival, synaptic plasticity, and neurogenesis processes (Vecchio et al., 2018), known to mostly take place at the hippocampal region, rather than the hypothalamus. This is consistent with AKT activation at the hippocampus, and not the hypothalamus. mTOR is activated by AKT and induces pS6 levels, while reducing the LC3BII/LC3BI ratio, respectively. In line with this, the levels of pS6 were elevated, and the LC3BII/LC3BI ratio was decreased in the hippocampus. Yet, phosphorylated AMPK, an mTOR inhibitor, was increased in the hippocampus of exercised mice compared to the control (Figures 4D, 5B). This suggests that another, as yet unknown, mechanism dissociates the effect of AMPK on mTOR or directly inhibits AMPK, regardless the increase in its phosphorylation. In support of this, uncoupling is the decrease in NRF2 expression under TM exercise in the hippocampus. This indicates that despite the increase in pAMPK, there is no activation of the mitochondrial biogenesis pathway (Figure 4B). The hypothalamus is a critical brain region that connects the neuroendocrine system to physiological functions (Sainsbury et al., 2002; Dietrich and Horvath, 2013). It is the metabolic center of the brain controlling energy homeostasis, glucose metabolism, and food intake (Roh and Kim, 2016). Therefore, activation of the AMPK signaling pathway observed in the hypothalamus can be explained by the known role of AMPK under low energy levels and the need for energy regulation under hypothalamic control (Oh et al., 2016). Indeed, along with this, mTOR inhibition was accompanied by reduced pS6 and increased autophagy levels. Thus, the signaling cascade activation in response to physical exercise is differently regulated in the hypothalamus and hippocampus, apparently due to their different regulatory functions (Roh and Kim, 2016; Roh et al., 2016).

While the different responses of the hippocampus and the hypothalamus to physical exercise can be explained by their different regulatory functions, metabolic tissues are thought to share similar responses to physical exercise. Strikingly, a comparison of gene expression between muscles and the liver, a metabolic tissue that regulates carbohydrate, lipid, and protein metabolism, revealed a different signaling cascade pathway in response to prolonged RW and TM exercise (Supplementary Figure S3A) (Mitra and Metcalf, 2012). In addition, unlike the gastrocnemius muscle, the quadriceps muscle, a different type of skeletal muscle, showed a different pattern of signaling cascade activity under physical exercise conditions (Supplementary Figure S2). Different types of skeletal muscles are composed of different myofiber types based on their different specified functions (Hawley et al., 2014). While slow twitch fibers are rich in the mitochondrial content and have high resistance to fatigue, fast twitch fibers have a high rate of glycolysis and low resistance to fatigue; thus, endurance exercise increases the amount of slow twitch fibers. Indeed, gastrocnemius was shown to have equal composition of slow and fast fibers, while the quadriceps muscle was shown to have more fast fibers than slow fibers. Thus, the differences between muscle types in gene expression could be explained by the differential effect of physical exercise on fiber type composition; each muscle type could change this fiber composition in response to exercise and lead to different responses. Overall, not only do those metabolic tissues not respond equally to endurance exercise but within the same metabolic tissue, different compositions will lead to a different responses (Wilson et al., 2012).

Above all, it is highly important to understand why the hypothalamus and gastrocnemius muscle activate similar signaling cascades in response to physical exercise. This could be explained by the role of the skeletal muscle and hypothalamus in energy homeostasis. The brain and the skeletal muscle have the highest demand for energy (Berg et al., 2002). Thus, they require energy fuel that will allow the tissue to perform the required actions, such as prolonged exercise. Unlike other metabolic tissues, such as the liver and fat, which consume energy, the gastrocnemius muscle and the hypothalamus use glucose and ketone bodies for fuel supplied from the liver to generate energy (Berg et al., 2002). We suggest that given these common responses of the muscle and hypothalamus, they show a parallel signaling cascade in response to physical activity.

Normal mitochondrial function is crucial for a healthier life span (Brand et al., 2013; Jang et al., 2018). Autophagy plays a major role in the maintenance and balance of protein homeostasis of mitochondria. This function was expressed in the hypothalamus under low energetic levels that led to the activation of the autophagic process and the formation of new mitochondria (Meng and Cai, 2011; Fujikawa et al., 2017). Similarly, prolonged physical activity also results in low energetic levels, which would also require the activation of the autophagic process and the formation of new mitochondria. Indeed, as shown in Figure 6, increased hypothalamic autophagy was found in groups subjected to both physical activities. These findings emphasize the importance of normal hypothalamic mitochondrial activity and the autophagic process of forming new mitochondria for the regulation of physiological functions and whole-body energy homeostasis and activation. Moreover, it emphasizes the importance of the hypothalamus as a regulator of physical exercise performance (Dietrich and Horvath, 2013; Kim et al., 2013; Roh and Kim, 2016; Roh et al., 2016).

Regardless of the tissue type, the type and duration of exercise were also important for determining the activated signaling. First, in order to observe physiological changes, a prolonged training program, of more than 10 days, is required. Second, it was previously shown that strength training specifically leads to an increase in muscle mass and an increase in the activity of the AKT-mTOR signaling pathway (Hawley et al., 2014). Here, we show that TM activates the AKT-mTOR pathway, suggesting that despite the common characterization of this activity as endurance, it can be considered a strength activity. Yet, surprisingly TM also promotes mitochondrial biogenesis by activating the AMPK-PGC1α signaling pathway, which is believed to negatively regulate mTOR. Hence, we suggest considering TM as a strength/endurance hybrid activity, and therefore, the changes were seen under forced TM exercise in both skeletal muscles and the hypothalamus, are consistent with this type of exercise.

SIRT6 was shown to negatively regulate AKT and mTOR and to activate AMPK (Kanfi et al., 2012). Yet, here, after physical training that induced AMPK activity, SIRT6 levels were reduced in the muscles. Moreover, the levels of the SIRT6 substrate, acK56, on histone H3 were increased. These findings suggest that the activation of AMPK in young mice is SIRT6-independent. However, hypothalamic histone H3 acK56 levels were significantly reduced. Thus, we suggest that SIRT6 activity, and not its levels, increased under both physical activities (Figures 5H, 6H), and this can explain the observed increased AMPK activity and autophagy in this tissue. Moreover, SIRT6 may regulate the beneficial effect of exercise in the hypothalamus, which subsequently controls the entirety of the body's responses to physical exercise, improving exercise performance (Roichman et al., 2021).

Altogether, this study explored the molecular metabolic response to physical activity in multiple tissues and demonstrates similar responses between the hypothalamus and muscles. It also suggests that like muscles, it might be possible to train the brain response by recurring exercises. This concerted molecular response enhances the coordination within the muscle–brain axis and potentially improves the systemic response to physical activity performance.

## Methods

### Animals, Housing Conditions, and Training Protocol

C57BL/6J male mice aged 4–6 months were kept under specific pathogen-free and 12-h day/night conditions with reverse light/dark cycle conditions with free access to a standard chow diet and water. The mice were randomly assigned to their treatment group and housed separately according to their chosen treatment group. All experiments were conducted in accordance with the Institutional Animal Care and Use Committee. The first experiment included three groups: control (without exercise), voluntary exercise using a running wheel, and forced exercise using a treadmill, with each group included six mice. For the short-term experiment, two different groups were included, with three mice in each group, control (without exercise) and forced exercise using a treadmill.

### Metabolic Cages and Treadmill

For this study, 4–6-month-old male mice were housed individually in TSE PhenoMaster System metabolic cages/treadmill and were allowed to acclimate for 5 days. For the voluntary exercise, experiment mice were housed individually for 10 h a day in the metabolic cage system with a running wheel for 30 days and housed back to their home cage in the end of the day. For the forced exercise using a one-lane treadmill, the animals were acclimated for 5 days for 10–30 min at a speed of 12 m/min before experimentation. The running speed was set to 12 m/min for 30 min, 5 days a week for 4 weeks, or until the mouse demonstrated fatigue, defined by an inability to move along the treadmill with stimulation (Pincu et al., 2016; Shin et al., 2016; Liu et al., 2017).

### Fat Mass Examination

Mice were measured for body composition in an NMR machine, and lean and fat mass were recorded as previously published (Roichman et al., 2016). Results presented demonstrate the combined data of each group: sedentary and forced exercise. In addition, once a week, all mice were weighed at same time and day.

### Tissue Harvest

For tissue collection, mice were killed 1 h or 24 h after the last exercise session by CO_2_ 4-h fasting in the first trial and 2-h fasting in the second trial. The liver, gastrocnemius muscle, quadriceps muscle, hypothalamus, hippocampus, and pre-frontal cortex were isolated, snap-frozen in liquid nitrogen, and then stored in −80°C until farther analysis.

### Gene Expression Analysis

For RNA extraction, the frozen liver, hippocampus gastrocnemius muscle, and quadriceps muscle tissues were homogenized in TRI reagent (Sigma) according to the manufacturer's specifications. RNA purity and concentration were assessed using a Thermo Scientific NanoDrop 2000 spectrophotometer (Thermo Scientific Nanodrop, USA), and 1 μg was taken for cDNA synthesis (first-strand cDNA synthesis kit, MBI Fermentas). Neuropeptide mRNA was analyzed using quantitative PCR carried out using SYBR green, fluorescent dye (Thermo Fisher Scientific) in a 15-μl total volume reaction containing 3 μl of cDNA. PCR amplification was performed with a CFX instrument (Bio-Rad). The following primers were used (Table 1).

**Table 1:** Primer sequences.

**Gene**	**Forward (5 → 3)**	**Reverse (3 → 5)**
SIRT6	CCTGTAGAGGGGAGCTGAGA	CCTGGCGGTCATGTTTTGT
SIRT1	AATGTGAGGAGTCAGCACCG	ACATTACCACGTCTGCAGCA
PGC1α	CCGAGAATTCATGGAGCAAT	TTTCTGTGGGTTTGGTGTGA
tFAM	CGGCAGAGACGGTTAAAAA	GCTGAACGAGGTCTTTTTGG
NRF2	GCAATGTGAGAGCAGGTTCA	GGAAATGCTTCCCTCCTTTC
Bdnf	GGACTTGTACACTTCCCGGG	ATGTTTGCGGCATCCAGGTA
Actin	AGCCATGTACGTAGCCATCC	CTCTCAGCTGTGGTGGTGAA

The expression levels of the liver, hippocampus, gastrocnemius muscle, and quadriceps muscle genes were measured in 4–6-month-old mice.

### Protein Expression Analysis

For protein extraction, frozen parts of the liver, gastrocnemius muscle, quadriceps muscle, hypothalamus, and hippocampus tissue samples were homogenized according to manufacturer's specification in urea buffer containing Tris pH 7.4 10mM, 150 mM NaCl, EDTA 1mM, EGTA 1mM, 0.5% Triton^*^100, urea 7M, and MEF lysis buffer were used for liver tissue containing Tris–HCl pH 7.4 1M, NaCl 5M, Triton^*^100, 0.5 % NP40, 10% glycerol, protease inhibitor, and phosphatase inhibitor were added to both lysis buffer. Protein levels were measured using the BCA method, and the protein extract was divided into aliquots and stored at −80°C.

### Western Blot

Totally, 10–20 μg of protein extracts were electrophoresed on 8–15% (v/v) polyacrylamide SDS-PAGE gels. The proteins were electrotransferred onto nitrocellulose membranes. The membranes were subsequently blocked, and after blocking, nitrocellulose membranes were incubated at room temperature with antibodies (1:1,000 #ab12486s cell signal SIRT6, 1:1,000 #ab5831s cell signal AMPK, 1:000 #ab2535s cell signal pAMPK,1:1,000 ab#4691s cell signal AKT, 1:1,000 #ab40605 pAKT (s473), 1:000 #07131 Millipore SIRT1, 1:1,000 #ab2217s S6 cell signaling, 1:1,000 #ab2211s pS6 cell signaling, 1:1,000 #ab2775 LC3B cell signaling 1:1,000 #ab4243 H3K56(Ac) abcam, 1:1,000 #ab70550 H3 abcam, 1:1,000 #ab10158 H4 abcam, 1:000 #ab47778 Actin Santa Cruz actin, 1:1,000 #abT5168 tubulin sigma). After incubation with primary antibodies, the membranes were washed in TBST^*^1 and incubated with secondary antibodies. After incubation with secondary antibodies, the membranes were repeatedly washed with TBST^*^1. The membranes were incubated with an ECL reagent, and protein bands were visualized. The bands were quantified by ImageJ software and shown as integrated density values.

### Phosphorylation/Acetylation Level Analyses

To measure phosphorylation/acetylation levels, the intensity of the phospho/acetyl-antibody was divided by the intensity of the protein antibody. To measure protein levels, protein intensities were divided by the intensity of the loading control.

### Quantification of Mitochondrial DNA

Total DNA was isolated by standard proteinase K and phenol–chloroform methods. The copy number of mtDNA was analyzed by quantitative real-time PCR. Of the total DNA, 0.4 ng was used as a template for the amplification of mtDNA. Product levels from primers against mouse mtDNA (5′-AAGACACCTTGCCTAGCCACAC-3′ and 5′-TGGCTGGCACGAAATTTACC-3′) were normalized to nuclear 18S rRNA gene (5′-AACTTTCGATGGTAGTCGCCG-3′ and 5′-CCTTGGATGTGGTAGCCGTTT-3′).

### Statistical Analysis

Two-tailed Student's *t*-test was used to compare differences between samples analyzed. All data are presented as means ± standard error of the mean. A *P-*value of 0.05 (*p* ≤ 0.05) was considered to be a statistically significant difference. The data obtained from measuring physiological parameters, gene expression, and protein extraction from the second trail were analyzed by two-way ANOVA, followed by Tukey or Bonferroni *post-hoc* test correct for multiple comparison.

## Data Availability Statement

The original contributions presented in the study are included in the article/Supplementary Material, further inquiries can be directed to the corresponding author/s.

## Ethics Statement

This study was reviewed and approved by the Bar-Ilan University Ethics Committee. All experiments were conducted in accordance with Bar-Ilan Institutional Animal Care and Use Committee and approved by the Ministry of Health of Israel.

## Author Contributions

HYC and AK conceived and designed the experiments and wrote the manuscript. AK, MG, YS, AR, and BL performed the experiments. All authors contributed to the article and approved the submitted version.

## Funding

This study was supported by the Israel Science Foundation (890/21 and 777/16), I-CORE (41/11), the U. S. Israel Binational Science Foundation (BSF, 2019312), and the SAGOL Center of Healthy Human Longevity.

## Conflict of Interest

The authors declare that the research was conducted in the absence of any commercial or financial relationships that could be construed as a potential conflict of interest.

## Publisher's Note

All claims expressed in this article are solely those of the authors and do not necessarily represent those of their affiliated organizations, or those of the publisher, the editors and the reviewers. Any product that may be evaluated in this article, or claim that may be made by its manufacturer, is not guaranteed or endorsed by the publisher.
