# Supplementary material for: Hypothalamus–Muscle Parallel Induction of Metabolic Pathways Following Physical Exercise
Source: Front Neurosci. 2022 Jul 19;16:897005. doi: 10.3389/fnins.2022.897005 (PMC9344923; doi:10.3389/fnins.2022.897005)
Supplement: Supplementary file 1 [file Data_Sheet_1.docx]

**Supplementary figures**

**Figure S1. Short Term endurance exercise is not sufficient to effect various physiological parameters.**

(A) Schematic figure of experimental design. (B) Body fat (g) of young (3-month-old) WT male mice from each group (control and treadmill). (C) Epididymal WAT weight at the end of the training session in young (4-6 month-old) WT male mice from each group control (CT), running wheel (RW) and treadmill (TM). The values shown are mean ± s.e.m., *, p<0.05; **,p<0.01; (two-way ANOVA followed by Tukey post-test adjusted for multiple comparisons for weight; and two-tailed t-test for and fat (%); n=6 for control 24h (CT 24H) and treadmill 24h (TM 24H); n=6 for control (CT), running wheel (RW) and treadmill (TM).

**Figure S2. Different types of skeletal muscle are affected differently by forced and voluntary exercise.**

Relative mRNA levels of (A) *Sirt1*, (B) *Sirt6* and (C-D) genes involved in mitochondrial biogenesis, (C) *Nrf2*, (D) *tFAM,* at rest (CT), or under voluntary (RW) and forced (TM) long term exercise in young (4-6 month-old) WT male mice. (E) Quadriceps muscle relative mRNA levels of *Sirt6*, *Sirt1* and *Pgc1α*, *tFAM* an*d* *Nrf2* genes involved in mitochondrial biogenesis, at rest (CT), or after short term forced (TM) exercise in young (4-6 month) WT male mice. (F) Gastrocnemius mtDNA content at rest (CT) or after long term voluntary (RW) or forced (TM) exercise. (G) Quadriceps muscle mtDNA content at rest (CT) or after short term forced (TM) exercise. The values shown are mean ± s.e.m., *, p<0.05; **, p<0.01; (two-way ANOVA followed by Tukey post-test adjusted for multiple comparison); In (A-D and F) n=5-6; CT, RW and TM. In (E and G) n=3 for CT and TM.

**Figure S3. The effect of forced and voluntary exercise on the liver tissue.**

A) Liver relative mRNA levels of *Sirt1*, *Sirt6* and *Pgc1α*, in addition to genes involved in mitochondrial biogenesis *Nrf2* and *tFAM*, at rest or after long term voluntary (RW) and forced (TM) exercise, in young (4-6 month-old) WT male mice. (B) Liver mtDNA content at rest or after long term voluntary (RW) and forced (TM) exercise. The values shown are mean ± s.e.m.; *, p<0.05 (two-way ANOVA followed by Tukey post-test adjusted for multiple comparison); n=6 CT, RW and TM.
