# Supplementary figures and images for: Hypothalamus–Muscle Parallel Induction of Metabolic Pathways Following Physical Exercise
Source: Front Neurosci. 2022 Jul 19;16:897005. doi: 10.3389/fnins.2022.897005 (PMC9344923; doi:10.3389/fnins.2022.897005)

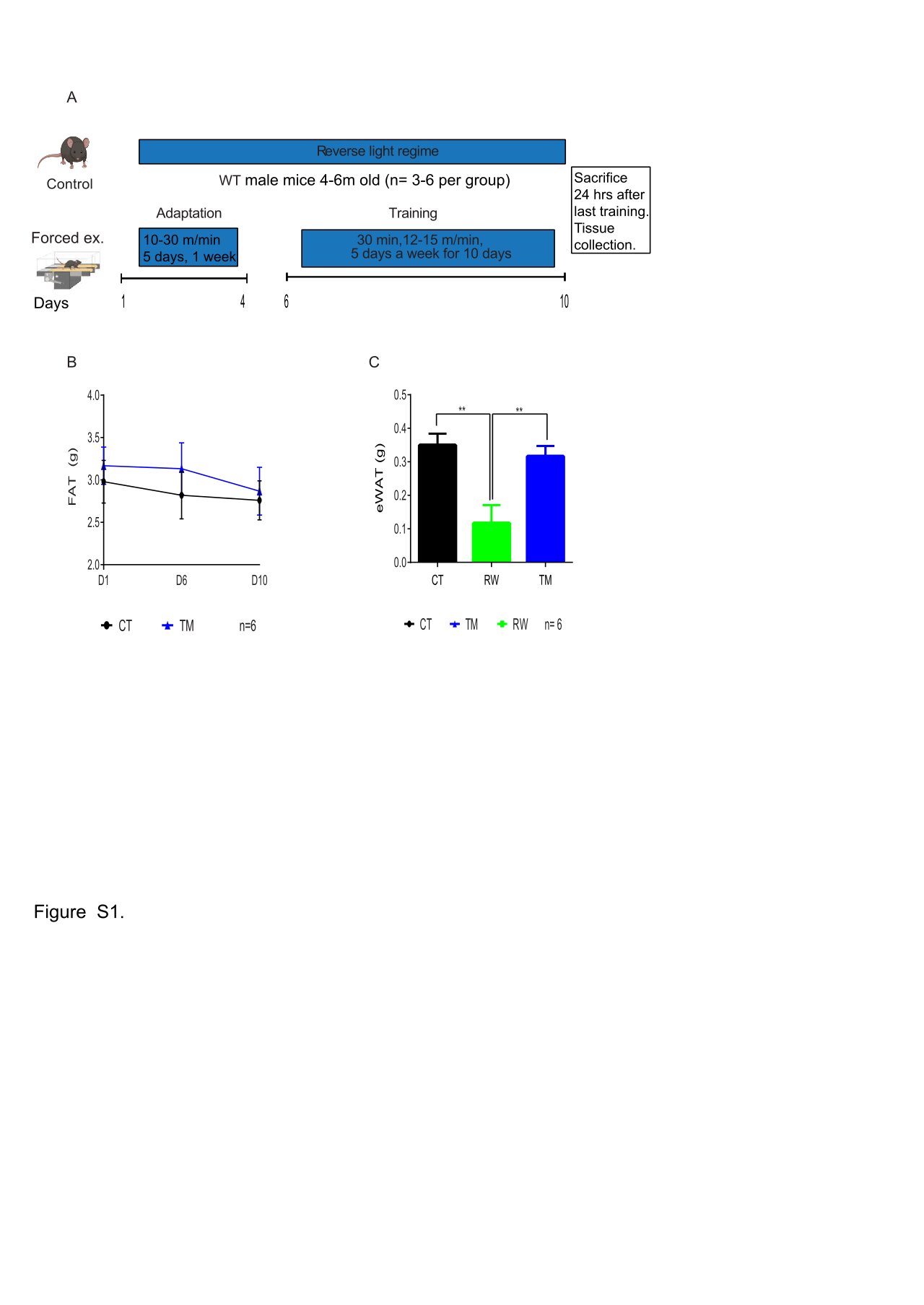

Supplement: Supplementary file 2 [file Image_1.jpg]

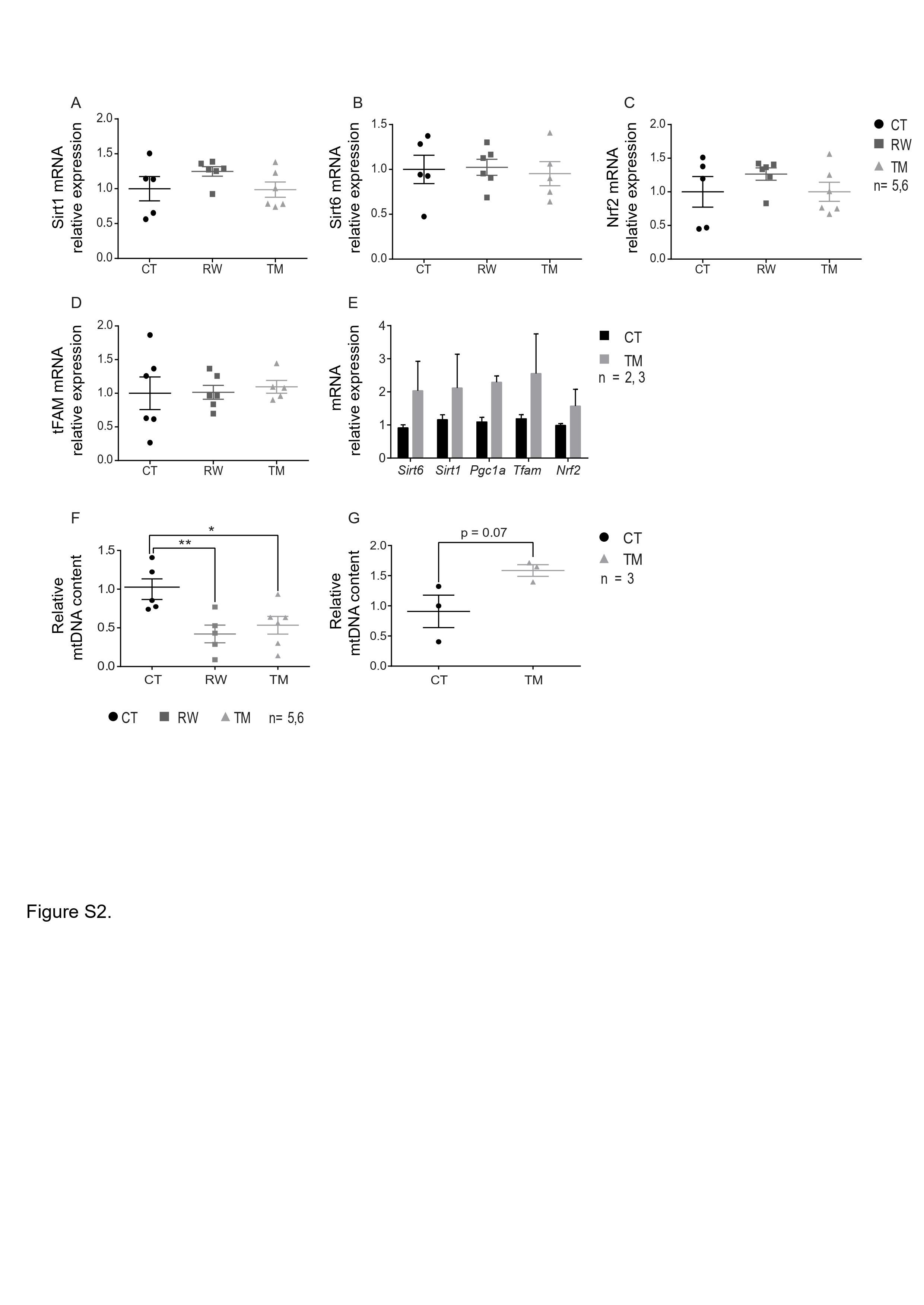

Supplement: Supplementary file 3 [file Image_2.jpg]

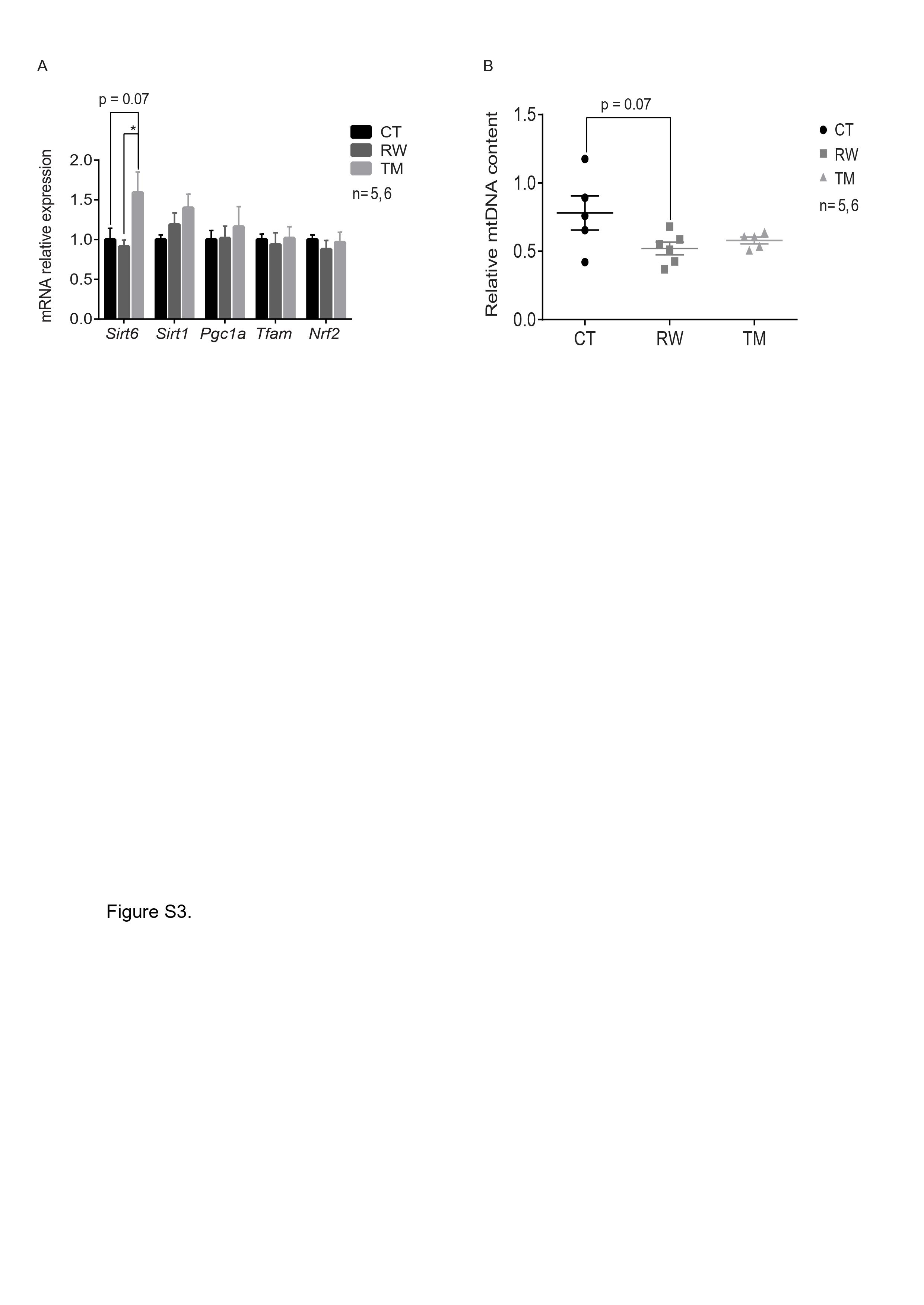

Supplement: Supplementary file 4 [file Image_3.jpg]
